# Supplementary material for: Improving HIV Outcomes in Miami’s Black populations with clinic-based community health workers protocol: The integrated navigation and support for treatment adherence, counseling, and research (INSTACARE) randomized controlled trial
Source: medRxiv. 2024 Dec 12:2024.12.11.24318859. Preprint. [Version 1] doi: 10.1101/2024.12.11.24318859 (PMC11661356; doi:10.1101/2024.12.11.24318859)
Supplement: 1 [file NIHPP2024.12.11.24318859V1-supplement-1.pdf]

medRxiv preprint doi: https://doi.org/10.1101/2024.12.11.24318859; this version posted December 12, 2024. The copyright holder for this preprint (which was not certified by peer review) is the author/funder, who has granted medRxiv a license to display the preprint in perpetuity.

It is made available under a CC-BY 4.0 International license .

SUPPORTING INFORMATION

S1 FIG. SPIRIT FIGURE

S2 TABLE. SPIRIT CHECKLIST

S1 FILE. INSTACARE PROTOCOL

Trial registration: ClinicalTrials.gov NCT04663152

Short title: Improving HIV outcomes in Miami's Black populations with clinic-based community health workers

INSTACARE SPIRIT 2013 Figure

	STUDY PERIOD			
	**Enrollment**	**Allocation**	**Post-allocation (Follow-up)**	**Close-out**
**Timepoint**	Month 0	Month 0	Months 1-12	Month 12
**Enrollment**				
Informed Consent	X			
Baseline Assessment		X		
**Intervention**				
Randomization		X		
CHW Intervention			X (up to 12 months)	
Usual Care			X	
**Assessments**				
Viral Load Testing (Primary Outcome)			X (every 3 months)	X
ART Adherence (ACTG Adherence Questionnaire)		X	X (6 months, 12 months)	X
Non-HIV Medical Care (Medical Records)			X (continuous)	
Hospitalizations (Medical Records)			X (continuous)	
Health-Related Social Needs (HRSN)		X	X (6 months, 12 months)	X
Racism Index (IRRS-B)		X	X (6 months, 12 months)	X
Food Security (HFIAS)		X	X (6 months, 12 months)	X
Doctor-Patient Interaction		X	X (6 months, 12 months)	X
Clinic HIV Stigma (Perceived Discrimination Scale)		X	X (6 months, 12 months)	X
Community HIV Stigma (Short Version Scale)		X	X (6 months, 12 months)	X
Social Support Assessment		X	X (6 months, 12 months)	X
Quality of Life (MOS-HIV Survey)		X	X (6 months, 12 months)	X
Self-Efficacy (HIV-ASES)		X	X (6 months, 12 months)	X
Empowerment (Health Empowerment Inventory)		X	X (6 months, 12 months)	X
Adverse Event Monitoring			Continuous	
Participant Compensation			$125 (enrollment, exit); $50 (3-, 6-, 9-month assessments)	

Spirit Figure 1
